# *In Vitro* and *In Vivo* Activities of Essential Oil from the Seed of *Anethum graveolens* L. against *Candida* spp.

**DOI:** 10.1155/2011/659704

**Published:** 2011-05-11

**Authors:** Hong Zeng, Jun Tian, Yuechen Zheng, Xiaoquan Ban, Jingsi Zeng, Yehong Mao, Youwei Wang

**Affiliations:** ^1^The State Key Laboratory of Agricultural Microbiology, Huazhong Agricultural University, Wuhan 430070, China; ^2^Key Laboratory of Combinatorial Biosynthesis and Drug Discovery (Wuhan University), Ministry of Education and Institute of TCM & Natural Products, Wuhan University School of Pharmaceutical Sciences, Wuhan 430071, China; ^3^Department of Dermatology, Affiliated Union Hospital, Tongji Medical College, Huazhong University of Science and Technology, Wuhan 430022, China

## Abstract

The essential oil produced from the seed of *Anethum graveolens* L. (Umbelliferae) was tested *in vitro* and *in vivo* anti-*Candida* activity. The microbroth dilution method was used in the minimal inhibitory concentration (MIC), according to M27-A3 of the guidelines of the Clinical and Laboratory Standard Institute (CLSI). And then, efficacy evaluation of essential oil in the prophylaxis and treatment of experimental vaginal candidiasis was performed in immunosuppressed mice. The anti-*Candida *activity was analyzed by microbiological and histological techniques and was compared with that of fluconazole (FCZ). The results showed essential oil was active *in vitro* against all tested strains, with MICs ranging from 0.312 *μ*L/mL (for *C. tropicalis, C. parapsilosis, and C. krusei*) to 0.625 *μ*L/mL (for 6 isolated *C. albicans* strains). Essential oil (2% v/v) was highly efficacious in accelerating *C. albicans* 09-1555 clearance from experimentally infected mice vagina by prophylaxis and therapeutic treatments. In both therapeutic efficacy and prophylaxis studies, the histological findings confirmed the microbiological results. The experimental results revealed that the tested essential oil is effective against vulvovaginal candidiasis in immunosuppressed mice.

## 1. Introduction

*Candida albicans* is the most common cause of opportunistic fungal disease in humans. In recent years, the nonalbicans *Candida* spp., such as *Candida tropicalis*, *Candida glabrata*, *Candida parapsilosis,* and *Candida krusei*, have also emerged as significant pathogens [1, 2]. Vulvovaginal candidosis (VVC) is one of the most common clinical manifestations of *Candida* spp., affecting 70–75% of women at least once in their lifetime [3]. There are several factors that can lead to the development of candidosis; these include immunodeficiency, endocrine disorders, and malignant diseases. Majority of the clinically used antifungals suffer from various drawbacks in terms of toxicity, drug-drug interactions, and lack of fungicidal efficacy, high cost, and emergence of resistant strains resulting from frequent usage [4, 5]. For example, amphotericin B is very toxic, while others, such as FCZ, are limited because of the high rate of primary and secondary resistance [6]. Despite the recent introduction of new antifungal drugs, these are still limited in number; hence, the great demand for novel antifungal agents justifies the intense search for new drugs that are more effective and less toxic than those already in use [7]. The essential oils from many plants are known to possess antiviral, insecticidal, and antioxidant properties as well as antibacterial and antifungal activities [8, 9].

*A. graveolens*, one of species of Umbelliferae, is a traditional Chinese herb. The essential oil obtained by steam distillation from their seeds were used early in the last century to treat many pathological conditions, such as disease of the uterus, cervical ectropion [10]. Some researchers have reported that essential oil from the seed of *A. graveolens*, a material not native to Xingjiang (China), possessed anti-*C. albicans* activity [11, 12]. However, to the best of our knowledge, the anti-*Candida* activity of essential oil from the seed of *A. graveolens* has not been demonstrated both *in vitro *and *in vivo. *Thus, the objective of the current research is to evaluate its anti-*Candida *activity *in vitro* and *in vivo* in order to develop new antifungal agents from natural products. 

## 2. Materials and Methods

### 2.1. Plant Materials

The seed of* A. graveolens* were procured from the Xinjiang Uighur Medical College, located in Hotan, Xinjiang, China. 

### 2.2. Essential Oil Isolation

The essential oil was obtained by steam distillation. The oil was dried over anhydrous Na_2_SO_4_ and preserved in a sealed vial at 4°C until further use. The yield of essential oil from the seed of* A. graveolens* was 3.5%.

### 2.3. Animals

Female BALB/c mice (22 ± 2 g) were obtained from the Laboratory Animal Center of Wuhan University (Wuhan, China). The photoperiods were adjusted daily to a cycle of 12 h of light and 12 h of darkness. The environmental temperature and relative humidity was constantly maintained at 21 ± 2°C and 50–70%, respectively. The mice were fed *ad libitum *on a diet of standard pellets and water. The study received clearance from the Institutional Animal Ethical Committee (IAEC) of the Committee for the Purpose of Control and Supervision of Experiments on Animals (CPCSEA), Wuhan University, Wuhan, China. 

### 2.4. Microorganisms

The 10 isolates of *Candida* studied in this work include *C. albicans* (*n* = 6), *C. tropicalis *(*n* = 1), *C. parapsilosis* (*n* = 1), and *C. krusei* (*n* = 1). All *Candida* species were clinically isolated from infected patients in the Department of Dermatology and Venereology of the Union Hospital located in Wuhan, China. The isolated positions of *Candida* species are presented in Table 1. All clinical isolates were identified according to morphology on corn meal agar, followed by germ tube formation, thick-walled spores, yeast spores, and assimilation-fermentation profiles in the API 20 system (bioMérieux, Marcy l'Étoile, France). For the *in vitro* experiment, all isolated strains were tested and yeast colonies were adjusted density of 1 × 10^3^ CFU/mL with haemocytometer. For the *in vivo* experiment, the* C. albicans* 09-1555 was used. It was isolated from vagina and adjusted density of 6 × 10^6^ CFU/mL with haemocytometer. 

### 2.5. *In Vitro* Susceptibility Tests

The broth macrodilution protocols based on the CLSI reference document M27-A3 [13] with modifications was used to determine minimal inhibitory concentration (MIC) for yeasts. 

Tests were performed in sterile 96-well plates, into which 100 *μ*L of RPMI-1640 (without sodium bicarbonate and L-glutamine at pH 7.0) were added to each well. Before inoculum, 100 *μ*L of the essential oil was added to the first well and serially diluted from the first well by taking 100 *μ*L into the second. This twofold dilution was continued down the plate, after which 100 *μ*L from the 10th column of the plate was discarded. The 11th column of the plate was reserved for negative control wells (without inoculation), and the last column was reserved for the positive growth control wells (without essential oil or FCZ). The essential oil and FCZ concentrations ranged from 20–0.039 *μ*L/mL and 100–0.18 *μ*g/mL, respectively. Then, the yeast colonies were suspended in RPMI 1640 medium and adjusted 1 × 10^3^ CFU/mL with haemocytometer (twice the final inoculum size), 100 *μ*L was added to each well of the 96-well plates.

The tests 96-well plates were incubated at 37°C for 48 h (*Candida* spp.), after which the MICs were determined. MICs were defined as the lowest concentration of the test substances that prevented visible growth of microorganisms. All experiments were performed in triplicate. 

### 2.6. *In Vivo* Activity of Essential Oil against Candida albicans 09-1555

The mice were maintained under pseudoestrus by giving them estradiol benzoate. These were then immunosuppressed by giving them dexamethasone, as previously reported by Martinez et al. [14]. In brief, the mice received estradiol benzoate on day 6 before inoculation (0.1 mg/20 g, once every two days, s.c.). They then received dexamethasone on day 1 before inoculation and on day 3 after inoculation (22.5 *μ*g/20 g, once a day, i.p.). On inoculation day, the mice were inoculated intravaginally with 6 × 10^6^ cells of *C. albicans* 09-1555 in 20 *μ*L. 

### 2.7. Prophylactic Treatment

Prior to the infection of the animals, they were separated randomly into 10 prophylactic groups (Groups P). Control (CK) (*n* = 10) was the negative control group that had neither infected nor treated animals. Group 1 (*n* = 10) had infected, untreated but not immunosuppressed animals; this group served to study the impact of immunosuppression on the development of the infection. Group 2 (*n* = 10) was the positive control group that had mice that were immunosuppressed and inoculated intravaginally with *C. albicans* 09-1555; this group received 20 *μ*L of excipient solution [1% sodium carboxymethylcellulose (CMC-Na), including 0.01% Tween 20] twice a day. Group P1 (*n* = 10) consisted of treated groups that had immunosuppressed and infected animals that received FCZ (20 *μ*L at 100 *μ*g/mL). Groups P2, P3, and P4 (*n* = 10 in each group) were the treated groups that had immunosuppressed and infected animals that received essential oils (20 *μ*L at 2, 1, and 0.0625% v/v). This treatment began 2 days before the inoculation of *C. albicans* 09-1555 in the vagina and continued 15 days thereafter at a dose of 20 *μ*L twice a day by intravaginal route. 

### 2.8. Therapeutic Treatment

Prior to inoculation, animals (*n* = 10) were separated randomly into 4 groups: Groups T1, T2, T3, and T4 (*n* = 10 in each group), all of which received therapeutic treatment with FCZ (100 *μ*g/mL) and essential oils (20 *μ*L at 2, 1, and 0.0625% v/v), respectively; all groups also received the same concentrations as the prophylaxis. This treatment began 4 day after the inoculation and continued for 15 days thereafter at a dose of 20 *μ*L twice a day by intravaginal route. These CK, Group 1 and Group 2 served as controls for both prophylactic and therapeutic treatments. 

### 2.9. The Microbiological Test

For the prophylactic treatment, the evaluation of vaginal burden was performed on samples washed with 1 mL of sterile saline buffer. The obtained cells were harvested by centrifugation at 3200 ×g for 15 min. This operation was repeated on days 2, 4, 8, 10, 12, and 15 after inoculation to observe the course of infection. Determination of the number of *Candida* organisms was conducted in duplicate after performing serial 10-fold dilution of washing fluid and plating on Sabouraud glucose agar containing 0.05% of chloramphenicol. All plates were incubated at 37°C for 24 h for each series of dilutions. For the therapeutic treatments, the evaluation of vaginal burden was carried out on days 4, 8, 10, 12, and 15 after-infection to observe the course of infection. 

### 2.10. The Histological Data

Vaginas were removed and longitudinally opened. These were then fixed in 10% formaldehyde solution for at least 48 h, stained using the periodic acid-Schiff (PAS) stain for fungal visualization. For the prophylactic treatment, the vaginas were removed on days 2, 8, and 15 after inoculation to observe the course of infection. For the therapeutic treatments, the vaginas were removed on days 4, 8, and 15 after-infection to observe the course of infection. 

### 2.11. Statistical Analysis

All experiments were done in triplicate, and the results were reported as mean ± S.E.M. (*n* = 6). The data were analyzed by one-way ANOVA. Statistically significant effects were further analyzed, and means were compared using Duncan's multiple range test. Statistical significance was determined at *P* < .01. 

## 3. Results

### 3.1. Antifungal Susceptibility Test

*In vitro *antifungal activity of essential oil was investigated against 10 clinical strains of yeasts. The MICs value is reported in Table 2. The results showed that the essential oil was active against all the tested strains. For *C. tropicalis, C. parapsilosis, *and *C. krusei *strains, MIC (0.312 *μ*L/mL) values were similar. For the 6 isolated *C. albicans* strains, MIC (0.625 *μ*L/mL) values were also the same. 

### 3.2. Microbiological Results of Prophylactic and Therapeutic Treatment

After establishing activity *in vitro*, low values of MICs were obtained, and we examined the activity of essential oil *in vivo*. The experimental model of the vaginal candidiasis model was used, and the anti-*Candida* activity of essential oil *in vivo *was evaluated.

**Figure 1:**
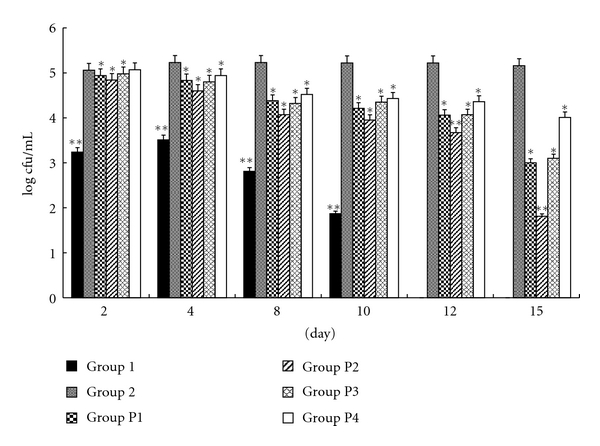
Microbiological study of the prophylactic efficacy of essential oil versus FCZ against vaginal candidiasis in mice. Outcome of vaginal infection by *C. albicans* 09-1555 in immunosuppressed BALB/c mice inoculated intravaginally with essential oil and FCZ. CK: negative control group (no *Candida* was found in the vaginal lumina). Group 1: infected, untreated but not immunosuppressed animals. Group 2: positive control group, immunosuppressed inoculated intravaginally with *C. albicans* 09-1555, received excipient. Group P1: treated groups: immunosuppressed, infected animals that received FCZ (20 *μ*L at 100 *μ*g/mL). Groups P2, P3, P4: treated groups: immunosuppressed, infected animals that received essential oils (20 *μ*L at 2, 1, and 0.0625% v/v). The value log cfu was showed by the mean ± S.E.M. (*n* = 6). **: compared with Group 2 (*P* < .01); *: compared with Group 2 (*P* < .05).

In the prophylactic treatment (Figure 1), prior to the initiation of the experiments, individual vaginal cavity cultures were performed and no *Candida* organisms were found. Essential oil and FCZ exerted a marked acceleration of the clearance of the yeast, as demonstrated by a statistically significant decrease in cfu counts 15 days after the vaginal challenge, compared with control Group 2. The clearance values with different essential oil concentrations suggest a substantial essential oil dose dependence of fungus clearance. As with all dose regimens, the infection was decreased in 15 days, whereas the untreated control mice remained infected (approximately log 5.16 ± 2.36 *C. albicans* cfu/mL of the vaginal fluid). In comparative terms, the acceleration of *Candida* clearance in mice treated with FCZ (100 *μ*g/mL) solution substantially overlapped the activity of a 1% v/v solution of essential oil. On the other hand, no effect on the rate of fungal clearance was observed in mice treated with essential oil-untreated animals, given the 1% CMC solution, including 0.01% Tween 20. The mice in CK (negative control group: neither infected nor treated animals) showed negative culture results throughout the experiment. Essential oil showed stronger anti-*Candida *activity than FCZ, similar to the results obtained *in vitro*. 

**Figure 2:**
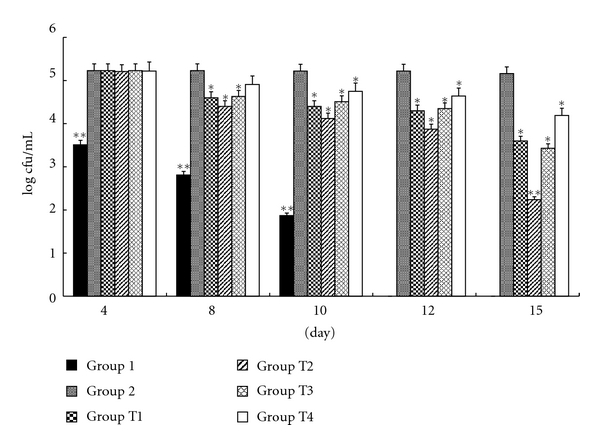
Microbiological study of the therapeutic efficacy of essential oil versus FCZ against vaginal candidiasis in mice. Outcome of vaginal infection by *C. albicans* 09-1555 in immunosuppressed BALB/c mice inoculated intravaginally with essential oil and FCZ. CK: negative control group (no *Candida* was found in the vaginal lumina). Group 1: infected, untreated but not immunosuppressed animals. Group 2: positive control group, immunosuppressed, inoculated intravaginally with *C. albicans* 09-1555, received excipient. Group T1: treated groups: immunosuppressed, infected animals that received FCZ (20 *μ*L at 100 *μ*g/mL). Groups T2, T3, T4: treated groups: immunosuppressed, infected animals that received essential oils (20 *μ*L at 2, 1, and 0.0625% v/v). The value log cfu was showed by the mean ± S.E.M. (*n* = 6). **: compared with Group 2 (*P* < .01); *: compared with Group 2 (*P* < .05).

In the therapeutic treatment (Figure 2), just before the inoculation with *Candida* cells, the vaginal cavities of all the mice were sampled and shown to be free from infection. In order to verify the establishment of the infection 4 days after inoculation, all the groups of animals were re-sampled. The results showed that the vaginal swabs were positive for all groups of animals.

After 15 consecutive days of therapeutic treatment (day 15), the viable *C. albicans* 09-1555 cells for FCZ (100 *μ*g/mL) and essential oils (2, 1, and 0.0625% v/v) treatments showed values of log 3.6 ± 1.77 and log 2.24 ± 1.93, 3.43 ± 1.25 and 4.19 ± 3 cfu/mL, respectively, indicating a significant reduction of *Candida* organisms compared with Group 2 with a value of 5.16 ± 2.36 cfu/mL (*P* < .01). However, there was no significant difference between FCZ (100 *μ*g/mL) and essential oil 1% (*P* > .05). CK showed negative culture results throughout the experiment. The infected animals in Group 1 (nonimmunosuppressed infected and untreated group) had no *Candida* cell. 

### 3.3. Histological Test

Prophylactic treatment: in all animals, the presence of yeast was found on sections of vaginas stained with PAS. In infected untreated mice, both budding yeast and the pseudohyphae form of *C. albicans* 09-1555—dark with PAS stain—were found in the luminal vagina. At the surface of the epithelium, we also noticed keratin debris, whereas in the noninfected control group, no *Candida* was observed in the luminal vagina.

**Figure 3:**
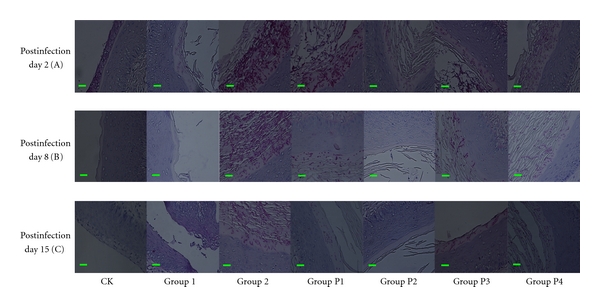
Microscopic observation of the prophylactic efficacy of essential oil versus FCZ against vaginal candidiasis in BALB/c mice on days 2, 8, and 15 after-infection (periodic acid-Schiff staining, 400x). CK: neither infected nor treated animals. Group 1: infected, untreated but not immunosuppressed animals. Group 2: immunosuppressed and inoculated intravaginally with *C. albicans* 09-1555, and animals that received excipient. Group P1: treated groups: immunosuppressed, infected animals that received FCZ (20 *μ*L at 100 *μ*g/mL). Groups P2, P3, P4: treated groups: immunosuppressed, infected animals that received essential oils (20 *μ*L at 2, 1, and 0.0625% v/v).The bar is 10 *μ*m.

The infected mice from Groups P1–P4 (immunosuppressed and then infected and treated with FCZ and essential oil) all showed budding yeast and pseudohyphae in the vaginal luminal, on day 2 after inoculation (Figure 3). However, those from Group 2 (immunosuppressed infected and treated with excipient) showed large amounts of yeast and pseudohyphae in the vaginal luminal (Figure 3). Other groups showed yeast and pseudohyphae in the vaginal luminal dose dependently. Samples from Groups P1, P2, P3, and P4 received FCZ (100 *μ*g/mL) and essential oils (2, 1, 0.0625% v/v) on day 8 after inoculation, less than that for *Candida* in the vaginal luminal (Figure 3), compared with Group 2 (Figure 3). On the 15th day after inoculation, Group P2 showed no *Candida* organisms detected by histological techniques (Figure 3) and it showed the same aspect as CK (neither infected nor treated animals), which was better than Group P1 (infected groups treated with FCZ).

In Group 1 (nonimmunosuppressed infected and untreated group), the infected animals had few pseudohyphae in the vaginal lumen on day 2 after inoculation, and no *Candida* was observed in the vaginal lumina on day 15. 

**Figure 4:**
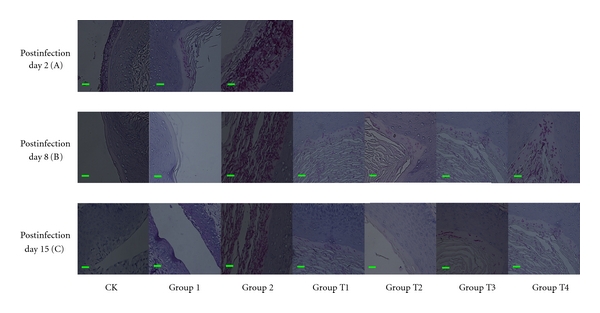
Microscopic observation of therapeutic efficacy of essential oil versus FCZ against vaginal candidiasis in BALB/c mice on Post-infection day 4, day 8, day 15 (periodic acid-Schiff staining, 400x). CK: neither infected nor treated animals. Group 1: infected, untreated but not immunosuppressed animals. Group 2: immunosuppressed, inoculated intravaginally with *C. albicans* 09-1555, received excipient. Group T1: treated groups:immunosuppressed, infected animals received FCZ (20 *μ*L at 100 *μ*g/mL). Groups T2, T3, T4 treated groups: immunosuppressed, infected animals received essential oils (20 *μ*L at 2% v/v, 1% v/v, 0.0625% v/v). The bar is 10 *μ*m.

Therapeutic treatment: vaginal sections of all the animals were also studied by light microscopy. Infected and untreated mice demonstrated the presence of *C. albicans* 09-1555 at the surface of the epithelium with desquamation of superficial layers, whereas in CK (neither infected nor treated animals), no *Candida* was found in the vaginal lumina (Figure 4). 

On day 4 after inoculation, the infected animals had great amounts of budding yeast and pseudohyphae in the vaginal lumina (Groups T1–T4 and Group 2) (Figure 4).

On day 8 after administration, therapeutic treatment with FCZ and essential oils (Groups T1–T4) significantly reduced the fungal burden compared with Group 2 (immunosuppressed infected and treated with excipient) in the vaginal luminal (Figure 4).

On the 15th day after administration, Group T2, (immunosuppressed infected and treated with essential oil 2% v/v) completely eradicated the vaginal *Candida* (Figure 4). Regarding Group T1 (immunosuppressed infected and treated with FCZ) (Figure 4), only a few *Candida* was found in the vaginal lumina similar to Group T3 (immunosuppressed infected and treated with essential oil 1% v/v).Vaginal sections in CK and Group1 presented the same aspect that no *Candida* was found in the vaginal lumina (Figure 4). 

## 4. Discussions

*A. graveolens* is found in many places, such as India, Europe, United States, Turkey, and China. It has been used for cooking and in Uygur medicine since ancient times in China. Aromatic herbal oils used for cooking and flavoring cover a broad spectrum of antimicrobial activities. The main chemical components of essential oil from the seed of *A. graveolens* are carvone and limonene [10]. These findings are similar to those reported in other studies [15, 16]. These activities may be attributed to the presence of the aforementioned main chemical components. Meanwhile, other authors have reported that carvone and limonene have antimicrobial activities [17]. 

 In this paper, we have confirmed and extended existing data on *in vitro *activity of essential oil against an elevated number of clinical isolates of *C. albicans* and other *Candida *species. The MIC values ranged from 1.56–3.15 *μ*g/mL for FCZ against *C. albicans* strains. The *C. albicans *09-1555 strain was nonsusceptible to FCZ. The MIC value was 3.15 *μ*g/mL. Another paper has reported that an FCZ MIC of ≥2 *μ*g/mL is considered nonsusceptible; it also reported a value of ≤2 *μ*g/mL as the FCZ susceptibility [18]. Yili et al. [11] have shown that the MIC value of essential oil from the seed of *A. graveolens* against *C. albicans* was 2.73 *μ*g/mL by broth dilution method. Although these results seem to contradict ours, we think this difference may depend on experimental conditions and material origin.

As far as the vaginal candidiasis model is concerned, the presence of estrogen is a very important factor for the persistence of experimental *Candidal* vaginitis [19, 20]. Our results showed that the control group of immunosuppressed, infected, and untreated animals remained infected throughout the experiment. In contrast, 15 days after inoculation, the group of nonimmunosuppressed, infected, and untreated mice showed spontaneous clearing of the yeast. These data confirmed that immunosuppression is necessary to the success of this model [14]. In this model, the antifungal activity of essential oil, which is being compared with that of FCZ, has been investigated and determined by microbiological tests and histological study. To the best of our knowledge, this is the first report showing the effect of essential oil from the seed of *A. graveolens* treatment on vaginal candidiasis. To allow optimal adhesion of essential oil and FCZ on the mucosal vagina, a gelatinous suspension of 1% CMC-Na was used as excipient to treat mice through the intravaginal route. Both 1% CMC-Na and 0.8% agar were used as excipients [20, 21]. On the basis of preliminary tests, 32-fold MIC of FCZ (*μ*g/mL) was chosen as a reference treatment; likewise, 2-fold MIC (0.0652% v/v), 32-fold MIC (1% v/v), and 64-fold MIC of essential oil (2% v/v) were chosen as reference treatments.

Some authors have already demonstrated the efficacy of azoles in vaginal candidiasis prophylaxis [22], while others have also demonstrated the efficacy of carvacrol and eugenol [20]. In this work, we investigated the efficacy of essential oil from the seed of *A. graveolens* by prophylactic treatment. For the* in vivo* results, 15 days after treatment, The mice treated with essential oils (2, 1, 0.0625% v/v) remained infected, with average *Candida* counts recorded as log 1.81 ± 1.4, 3.1 ± 1.23, 4.01 ± 2.73 cfu/mL, respectively. But they were significantly lower than those found in the infected untreated control group. Group 2 showed positive *Candida* cultures throughout the experiment. In Group P2 (the animals treated with essential oil 2% v/v), few animals showed negative culture on 15 days after the interruption of the treatment. The histological examination agreed with the microbiological results. Prophylactic treatments with FCZ, essential oil (1% v/v), and essential oil (0.0625% v/v) only seemed to be less effective than that with essential oil (2% v/v). 

Furthermore, vaginal burden in the remaining infected animals was significantly less than in the control group, and few *C. albicans* 09-1555 were detected on the vaginal sections. From these results, essential oil (2% v/v) was shown to be the most effective in preventing *Candida* vaginal infection.

Therapeutic treatment with essential oil (2% v/v) for 15 days led to significant clearance of yeast from the majority of animals exhibiting negative *Candida* culture compared with Group 2 (the infected untreated); however, few *Candida* cells were detected in one remaining infected mice by microbiological results, with no detectable *Candida* cells using histological examination. Concerning the animals treated with essential oil (1% v/v), essential oil (0.0625% v/v), few *Candida* cells were in the vaginal lumina detected by both microbiological and histological examinations. The results showed similar values for animals treated with FCZ was (100 *μ*g/mL) and with essential oil (1% v/v). The FCZ also used as positive control by other author. Although these results seem to contradict those of previous works, the difference may depend on experimental conditions, such as administration time, inoculum size, and different animal strains [23, 24]. Therefore, the therapeutic treatment with essential oil (2% v/v) was shown as the most effective in preventing *Candida* vaginal infection.

The results of our investigations demonstrate that essential oil treatment is efficacious in resolving experimental *Candida* infection. In the case of the nonsusceptible organism, treatment with essential oil was comparable to a standard treatment with FCZ. 

## 5. Conclusion

Based on these results, it can be concluded that essential oil from the seed of *A. graveolens* possesses anti-*Candida* activity* in vitro* and *in vivo*. These results support the traditional use of essential oil from the seed of *A. graveolens* in antimicrobial capabilities, and it could be considered as a kind of new parts for medicinal material which is useful and potential in prevention and treatment of monilial vaginitis. 

## Figures and Tables

**Table 1:** Isolated positions of the *Candida* species.

Strain	Number	Isolating position
*C. albican*	09-1519	Coronary sulcus
*C. albican*	09-1522	Throat swab
*C. albican*	09-1502	Oral mucosa
*C. albican*	09-1634	Dejecta
*C. albican*	09-1555	Vagina
*C. albican*	09-1394	Coronary sulcus
*C. krusei*	09-1681	Vagina
*C. tropicalis*	032	Throat swab
*C. parapsilosis*	07-305	Hand

**Table 2:** Antifungal activities of essential oil from the seed of *A. graveolens *against *Candida *spp.

Strain	Fluconazole (*μ*g/mL) MIC	Essential oil (*μ*L/mL) MIC
*C. albicans* 09-1519	3.125	0.625
*C. albicans* 09-1522	3.125	0.625
*C. albicans* 09-1502	1.56	0.625
*C. albicans* 09-1634	1.56	0.625
*C. albicans* 09-1555	3.125	0.625
*C. albicans* 09-1394	3.125	0.625
*C. krusei *09-1681	25	0.312
*C. parapsilosis *07-305	0.78	0.312
*C. tropicals *032	3.125	0.312
